# Comparison of Risk Factors, Their Interaction Patterns, and Scoring Systems for Liver Cancer Between Patients With and Those Without Diabetes: Retrospective Cohort Study Using Electronic Health Records and Tree-Structured Algorithms

**DOI:** 10.2196/72239

**Published:** 2025-10-27

**Authors:** Sarah Tsz Yui Yau, Chi Tim Hung, Eman Yee Man Leung, Albert Lee, Eng Kiong Yeoh

**Affiliations:** 1 JC School of Public Health and Primary Care, The Chinese University of Hong Kong, 4/F, School of Public Health Building, Prince of Wales Hospital, Shatin, Hong Kong, China (Hong Kong), 852 22528790

**Keywords:** liver cancer, diabetes, risk factor, interaction, risk prediction, risk score, risk stratification, decision tree, random forest, survival analysis

## Abstract

**Background:**

Patients with diabetes are at higher risk of developing liver cancer. Nevertheless, risk factors and their interaction patterns have rarely been compared between patients with and those without diabetes, nor have their interactions been incorporated into scoring system development.

**Objective:**

This study aims to compare risk factors, their interaction patterns, and resulting scoring systems for liver cancer risk according to diabetes and liver disease status using tree-structured algorithms.

**Methods:**

A retrospective cohort study was conducted using electronic health records in Hong Kong. Patients who had used public health care services between 1997 and 2021 without cancer history were identified and followed up until December 31, 2021. Scoring systems were developed based on aggregate results from individual survival trees in random survival forest, and interaction patterns among factors were separately examined using conditional inference survival tree.

**Results:**

Of the 190,971 patients included, 1275 developed liver cancer during follow-up (median 6.25 y). Across 4 scoring systems, alanine aminotransferase (ALT) levels, age, sex, and triglycerides were commonly chosen as predictors irrespective of diabetes and liver disease status. In the overall systems, liver cirrhosis was additionally selected as a predictor, with chronic viral hepatitis uniquely chosen in diabetes. In the absence of liver disease, fasting glucose and smoking were uniquely selected for diabetes and nondiabetes, respectively. Chronic viral hepatitis appeared as the strongest risk factor in diabetes but not in nondiabetes. Among people with diabetes, in the absence of chronic viral hepatitis, sex became the most important factor, followed by age, statin use, and ALT levels. Among people without diabetes, age became the most dominant risk factor. For older patients (>55 y), uncontrolled lipids and male sex became key risk factors in statin and nonstatin users, respectively, when the ALT level was higher (>43.4 U/L), while smoking became a key risk factor when the ALT level was lower (≤43.4 U/L). For younger patients (≤55 y), sex remained the most significant factor.

**Conclusions:**

Patients with and those without diabetes exhibit distinctive interaction patterns among key factors on liver cancer risk. The resulting scoring systems reflect interaction patterns among predictors in individual survival trees. This study may help identify targets for public health interventions and provide clinical cancer risk prediction according to diabetes status.

## Introduction

Liver cancer is the third leading cause of cancer death worldwide [1]. Previous research has shown that diabetes is associated with an increased risk of cancer at several sites, including the liver [2,3]. Nevertheless, there is a lack of systematic comparison on the interaction patterns among key factors between patients with and those without diabetes. Nor have scoring systems been developed separately according to diabetes status.

While most risk prediction models for liver cancer focus on patients with known risk factors such as liver cirrhosis, chronic viral hepatitis, hepatic steatosis, and heavy alcohol use [4,5], several risk scoring systems developed among patients with [6,7] and those without known risk factors [8] have incorporated diabetes as a predictor. Nevertheless, only a few risk scoring systems have been developed specifically for diabetes population [9,10]. Si et al [9] has developed a scoring system for liver cancer prediction among people with diabetes without chronic viral hepatitis or alcoholic cirrhosis from South Korea with only 3 predictors, namely age, triglycerides, and gamma-glutamyl transferase (GGT), where lower triglyceride levels were found to be predictive of an elevated risk of liver cancer. On the other hand, Li et al [10] have incorporated a number of predictors, including age, sex, smoking, alanine aminotransferase (ALT) levels, glycated hemoglobin, liver cirrhosis, chronic viral hepatitis, and lipid profile into a scoring system for liver cancer prediction developed among people with diabetes from Taiwan. However, these diabetes-specific scoring systems lack comparison with people without diabetes, nor were interaction patterns among covariates examined.

Existing scoring systems rarely incorporate interaction patterns into variable selection. Nevertheless, previous research suggests that factors associated with liver cancer are often interrelated. For example, while both chronic hepatitis B or C [11] and diabetes [3] are independent risk factors for liver cancer, previous studies have also shown that hepatitis B or C infection could be linked to an elevated risk of type 2 diabetes development [12,13]. Also, while sex-specific elevated ALT thresholds are available [8], the potential age dependence of ALT [14-16] has rarely been incorporated. Furthermore, despite the common coconsumption of alcohol and smoking, and heavy alcohol use as known cause for liver damage [11], the role of smoking in liver injury is less clear [17,18].

While Cox proportional hazards regression is conventionally applied to examine a time-to-event outcome, tree-structured (or recursive partitioning) algorithms are more suitable to handle interactions among covariates and nonlinearity between a set of covariates and an outcome. On one hand, single tree-structured algorithms are intuitively interpretable and could be visually illustrated to demonstrate the interaction patterns [19,20] among split variables. Unlike traditional tree-structured algorithms which often lack a theoretical foundation, conditional inference survival tree [21] is embedded with statistical theory of conditional inference. On the other hand, tree-structured ensemble algorithms tend to be more stable and their aggregate results have lower variance than results from individual trees. Recently, Xie et al [22] have proposed a framework to develop clinical scoring system by integrating random survival forest in variable selection and retaining the use of beta coefficients from Cox regression in generating a final scoring system, where interaction patterns among covariates are taken into account in scoring system development, and clinical interpretability is ensured in the final scoring system.

The objective of the study is to compare risk factors, their interaction patterns, and resulting scoring systems for liver cancer risk according to diabetes and liver disease status using tree-structured algorithms.

## Methods

### Study Design and Study Population

This is a retrospective cohort study performed using territory-wide electronic health records in Hong Kong. The Hospital Authority (HA) is a statutory body responsible for managing public health care services and maintains a centralized clinical data repository, which stores information on patient demographics (sex and year of birth), disease diagnoses, prescription records, laboratory measurements, clinical notes, and radiology reports. Data used in this study were linked to death records from the Immigration Department. Individual-level data across datasets were linked via pseudonymous identifiers. Disease diagnoses were coded according to the *ICD-10* (*International Statistical Classification of Diseases, Tenth Revision*), or the International Classification of Primary Care, 2nd Edition (ICPC-2). Data were accessed via HA Data Collaboration Lab.

### Patients

Patients who had used public health care services between 1997 and 2021 were initially identified. Laboratory records on (1) ALT levels, (2) fasting glucose, (3) low-density lipoprotein (LDL) cholesterol, (4) high-density lipoprotein (HDL) cholesterol, and (5) triglycerides were extracted. Patients with at least one record on each of the above laboratory measurements were selected. Prescription records on antidiabetic drugs were extracted to determine diabetes status of patients. Those who were prescribed any antidiabetic drugs during the study period were identified as patients with diabetes, while those who did not receive any antidiabetic drugs during the study period were identified as patients without diabetes. Patients with diabetes who had at least 2 records on each selected laboratory measurement within 1 year of initiation of antidiabetic drugs or diabetes onset were further selected (n=161,790). Patients without diabetes who had at least 2 records on each selected laboratory measurement within 1 year of the earliest record of the above measurement were further selected (n=67,777). For each laboratory measurement, the mean value of at least 2 records closest to the baseline within 1 year was taken as baseline measurement. The index dates for patients with and those without diabetes were initiation of antidiabetic drugs and earliest selected laboratory record, respectively. For both patients with and those without diabetes, those with a cancer history at baseline were excluded. To exclude possible cases of type 1 diabetes, patients who were diagnosed with diabetes younger than 30 years [23], or those diagnosed with diabetes younger than 60 years and received insulin treatment within 1 year of diabetes onset [23] (but did not receive any other antidiabetic drugs during the study period) were excluded. In addition, exclusion of early insulin users may help remove more severe diabetes cases, since we intend to capture an incident diabetes cohort. Patients without diabetes younger than 30 years at baseline were also excluded. For both patients with and those without diabetes, to minimize reverse causality, those with less than 6 months of follow-up were excluded [24]. In addition, since the diagnosis of one cancer may influence the diagnosis of another cancer [24], those who were diagnosed with cancer types other than liver cancer during follow-up were also excluded. In other words, only patients who developed liver cancer or remained cancer-free during follow-up were included. Finally, a total of 132,221 patients with diabetes and 58,750 patients without diabetes were included.

### Outcome

The outcome of interest was diagnosis of liver cancer (ICD-10: C22) during follow-up.

### Input Variables

The set of input variables included demographics (sex and age at baseline), disease history, lifestyle behavior (smoking), laboratory measurements, and medication use. Disease history included liver cirrhosis [11], chronic viral hepatitis [11], fatty liver [11], and common comorbidities (ischemic heart disease, cerebrovascular disease, heart failure, chronic obstructive pulmonary disease, pneumonia, tuberculosis, hematuria, and cystitis). Disease diagnoses were extracted from both inpatient and outpatient diagnosis codes. The presence of fatty liver was determined from diagnosis codes or radiology reports (ultrasonography, computed tomography, and magnetic resonance imaging). Smoking habits were extracted from clinical notes, and patients were categorized as ever versus never smokers. Laboratory measurements included fasting glucose, LDL cholesterol, HDL cholesterol, triglycerides, and ALT levels. Medication included antidiabetic drugs (metformin, sulfonylurea, insulin, dipeptidyl peptidase-4 inhibitors, acarbose, meglitinide, glitazone, sodium-glucose cotransporter-2 inhibitors, and glucagon-like peptide-1 receptor agonists), aspirin, nonsteroidal anti-inflammatory drugs, anticoagulants, antiplatelets, statins, antihypertensive drugs (alpha-blockers, angiotensin-converting enzyme inhibitors, angiotensin receptor blockers, beta-blockers, calcium channel blockers, and diuretics). Medication use was defined as whether patients had taken a drug at baseline.

### Data Analysis

#### Conditional Inference Survival Tree

Conditional inference survival tree [21] was applied to examine the interaction patterns among the set of candidate predictors. At each split, a global null hypothesis of independence between a set of covariates and an outcome is tested at a prespecified α level. If rejected, a set of partial null hypotheses of independence between each covariate and an outcome is then tested at the same α level. The covariate with the minimum Bonferroni-corrected *P* value smaller than α is then selected as the split variable. Partitioning is recursively conducted until the global null hypothesis cannot be rejected. For continuous variables, the cutoff point is the optimal value to maximize the between-group differences in survival probability. Each path from the root node to a terminal node represents an interaction pattern [20]. Two separate survival trees, which are independent of the random survival forest used in scoring, were generated according to diabetes status. The α and maximum depth of the survival trees were set at .01 and 4 respectively.

Advantages of this algorithm include (1) incorporating conditional inference framework into the partitioning procedures, (2) avoiding overfitting, (3) minimizing bias toward selecting covariates with many possible values, and (4) not requiring explicit pruning.

#### Scoring System Guided by Random Survival Forest

The scoring system development followed a previously proposed framework [22]. For each scoring system, patients were first split into 70% train, 10% validation, and 20% test sets by default. Variable importance was first ranked by random survival forest using the train set. Random survival forest is a tree-structured ensemble algorithm to produce aggregated results from a predetermined number of decorrelated individual survival trees. There are 2 random components in random survival forest, namely bootstrapping in sampling and random selection in feature selection. To build each tree, a bootstrapped train set is selected. At each split, a set of *k* number of predictors is selected from a full set of *m* number of predictors, where *k* is equal to the square root of *m* by convention. Considering computing time, the number of trees in the survival forest was set at 10. Each variable was then added to a Cox regression model sequentially according to their ranking in variable importance in the random survival forest. The performance of the model was then evaluated on the validation set using area under the curve as a metric. Variables for the final scoring system were then selected using model improvement and parsimony as criteria. For continuous variables, the cutoff points were determined by default quantiles. Fine-tuning of variable selection and cutoff points was conducted according to domain knowledge and existing literature. The chosen set of variables was then added into a final scoring system, where score assignment was based on beta coefficients in the Cox regression model and model performance was evaluated on the unseen test set using Harrell’s concordance index (C-index) and integrated Brier score as metrics. In total, 4 scoring systems were separately developed according to diabetes and liver disease (presence or absence of liver cirrhosis or chronic viral hepatitis) status.

 Advantages of this approach include (1) tree-structured algorithms have higher interpretability than other machine learning algorithms, (2) aggregate results from a tree ensemble may have lower variance than results from individual trees, (3) interactions among covariates on an outcome in single trees are taken into account in a tree ensemble, (4) adopting a more objective way to incorporating less established risk factors as predictors in variable selection process, (5) categorizing continuous variables in deriving scoring systems may help address nonlinearity, and (6) converting risk prediction models into point-based scores could be more clinically useful.

### Ethical Considerations

Ethics approval for secondary data analysis was provided by the Chinese University of Hong Kong – Survey and Behavioral Research Ethics Committee (reference number: SBRE-22‐0386). Patient consent was waived since individuals were not identifiable in this study.

## Results

### Overview

A total of 190,971 patients were included. Among patients with (n=132,221) and those without (n=58,750) diabetes, 954 and 321 developed liver cancer during follow-up (median 6.25 y), respectively. The corresponding incidence rates were 1.04 and 0.61 per 1000 person-years. Among 129,609 patients with diabetes and 58,635 patients without diabetes in the absence of liver disease, 792 and 310 developed liver cancer during follow-up, respectively.

### Interaction Patterns–Diabetes

**Figure 1.**
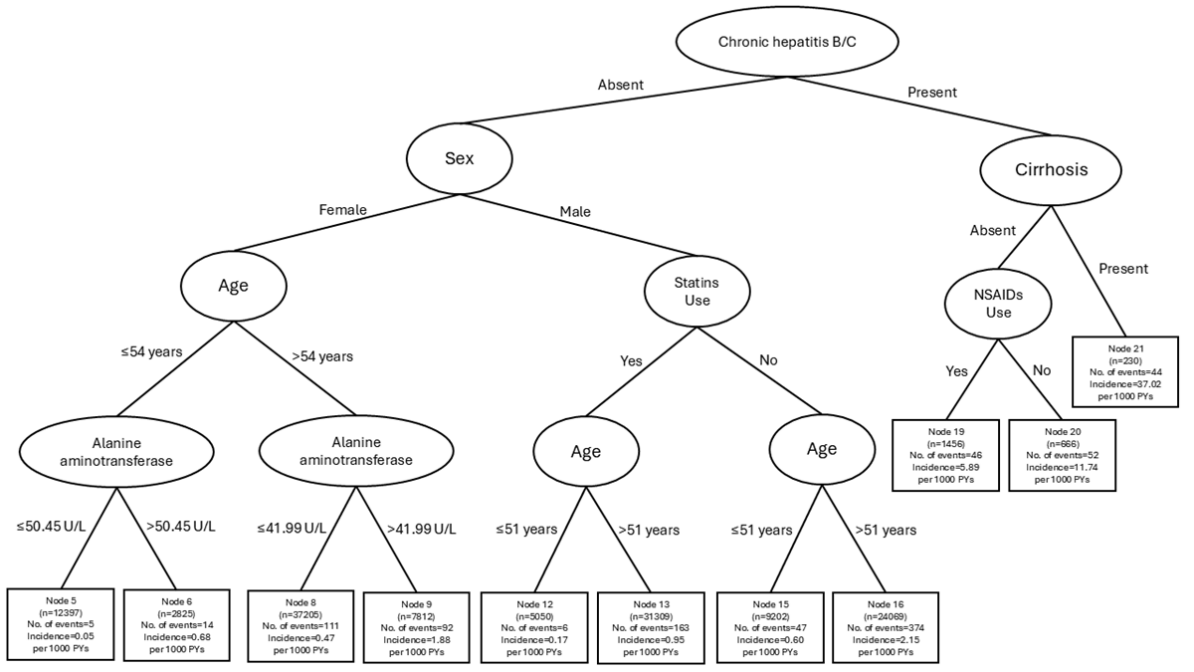
Conditional inference survival tree illustrating the interaction patterns among factors associated with the risk of liver cancer among patients with diabetes. NSAID: nonsteroidal anti-inflammatory drugs; PYs: person-years; U/L: unit per liter.

Chronic hepatitis B or C status emerged as primary factor in differentiating the risk of liver cancer. In the presence of chronic viral hepatitis, the most dominant risk factor was liver cirrhosis. On the other hand, in the absence of chronic viral hepatitis, sex became the most significant factor. In males, statin use and age were key factors. In females, age and ALT levels became key factors (Figure 1).

#### Absence of Chronic Viral Hepatitis, Male Sex, Statin Use, and Age

Among males in the absence of chronic viral hepatitis, statin use became the dominant factor in differentiating the risk of liver cancer. Across statin users and nonstatin users, an age of 51 years was symmetrically identified as optimal cutoff for differentiating liver cancer risk (Figure 1).

#### Absence of Chronic Viral Hepatitis, Female Sex, Age, and ALT Levels

Among females in the absence of chronic viral hepatitis, age became the subsequent dominant factor in differentiating the risk of liver cancer. Among older (>54 y) and younger (≤54 y) females, elevated ALT was identified as the most important risk factor at differential optimal cutoffs (41.99 and 50.45 U/L for older and younger females, respectively; Figure 1).

### Interaction Patterns—No Diabetes

**Figure 2.**
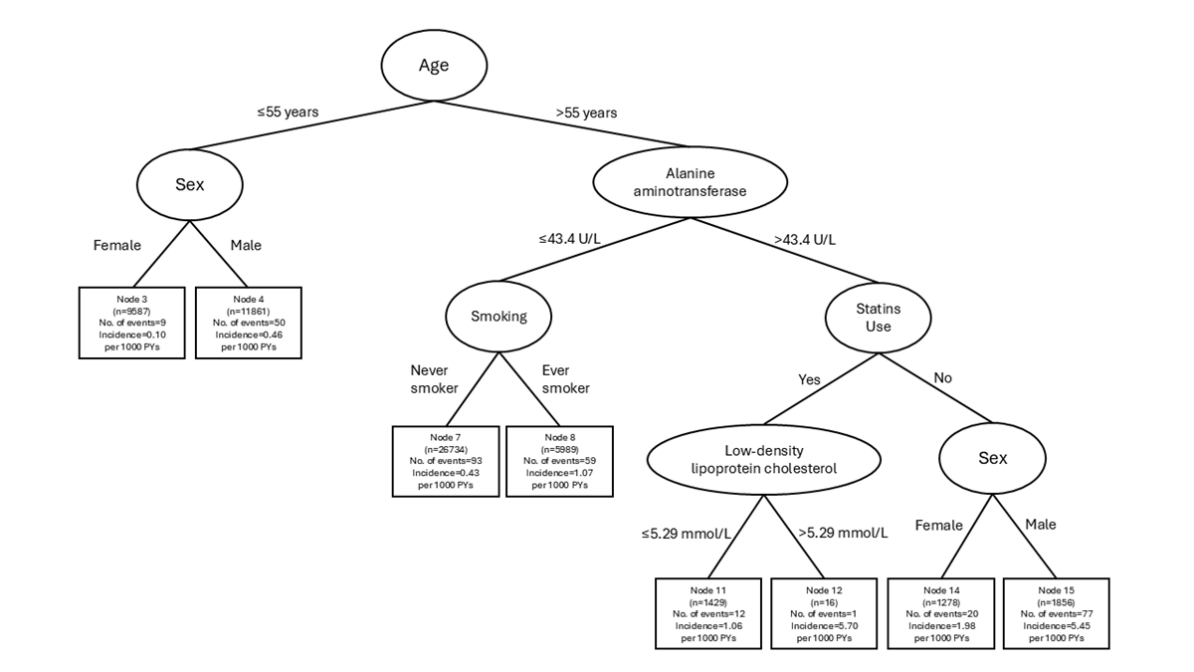
Conditional inference survival tree illustrating the interaction patterns among factors associated with the risk of liver cancer among patients without diabetes. PYs: person-years.

Age emerged as the primary factor in differentiating the risk of liver cancer. Among older patients (>55 y), ALT level became the dominant factor. For those with higher ALT levels, statin use, LDL cholesterol, and sex became key factors. For those with lower ALT levels, smoking became the dominant risk factor. Among younger patients (≤55 y), male sex became the most significant risk factor (Figure 2).

#### Older Age, Higher ALT Levels, Statin Use, and LDL Cholesterol or Sex

Among older patients (>55 y) with higher ALT levels (>43.4 U/L), uncontrolled LDL cholesterol (>5.29 mmol/L) and male sex became dominant risk factors in statin users and nonstatin users respectively (Figure 2).

#### Older Age, Lower ALT, and Smoking

Among older patients (>55 y) with lower ALT levels (≤43.4 U/L), smoking became the most important risk factor.

### Overall Scoring Systems by Diabetes Status

In the overall scoring systems, liver cirrhosis, ALT levels, age, sex, and serum triglycerides were commonly included as predictors regardless of diabetes status. Chronic hepatitis B or C status was uniquely included in diabetes but not in nondiabetes (Table 1). Comparing to the overall scoring system for diabetes-only (D_0_), generally, there was an increase in relative contribution of liver cirrhosis and triglycerides in the overall scoring system for nondiabetes-only (N_0_). Nevertheless, triglycerides exhibited an inverse association with the risk of liver cancer across diabetes (D_0_) and nondiabetes (N_0_) models (Table 2).

**Table 1.** Variable selection in scoring systems for liver cancer prediction among patients with and those without diabetes.

	Overall		No liver disease	
Variable	DM^a^ (D_0_)	Non-DM (N_0_)	DM (D_1_)	Non-DM (N_1_)
Chronic hepatitis B or C	✓			
Liver cirrhosis	✓	✓		
Alanine aminotransferase	✓	✓	✓	✓
Age	✓	✓	✓	✓
Sex	✓	✓	✓	✓
Triglycerides	✓	✓	✓	✓
Fasting glucose			✓	
Smoking				✓

a DM: diabetes mellitus.

**Table 2.** Scoring systems for liver cancer prediction among patients with and those without diabetes.

	DM^a^ (D_0_)		Non-DM (N_0_)	
Variable	Value	Point	Value	Point
Chronic hepatitis B or C				
	None	0	—^b^	—
	Chronic hepatitis B-only	13	—	—
	Chronic hepatitis C-only	19	—	—
	Coinfection of chronic hepatitis B and C	9	—	—
Alanine aminotransferase (U/L)				
	<11.5	0	<11	2
	[11.5, 17)	3	[11, 15.5)	0
	[17, 44.1)	9	[15.5, 38)	4
	[44.1, 83.1)	19	[38, 76)	16
	≥83.1	21	≥76	20
Age (years)				
	<42	0	<40	0
	[42, 51)	12	[40, 50)	14
	[51, 72)	24	[50, 71)	22
	≥72	27	≥71	30
Liver cirrhosis				
	Absent	0	Absent	0
	Present	16	Present	24
Sex				
	Female	0	Female	0
	Male	7	Male	8
Triglycerides (mmol/L)				
	<0.73	11	<0.875	18
	[0.73, 1.02)	9	[0.875, 1.88)	16
	[1.02, 2.26)	5	[1.88, 2.82)	14
	[2.26, 3.58)	0	≥2.82	0
	≥3.58	1	—	—

a DM: diabetes mellitus.

b Not applicable.

### Scoring Systems by Diabetes Status in the Absence of Liver Disease

In the absence of liver disease, ALT levels, age, sex, and triglycerides were commonly included as predictors irrespective of diabetes status. Baseline fasting glucose and smoking status were unique predictors in the scoring systems for diabetes (D_1_) and nondiabetes (N_1_), respectively (Table 1). The relative contributions of common predictors appeared comparable across 2 models (D_1_ and N_1_). In addition, triglycerides consistently demonstrated a negative relationship with the risk of liver cancer across diabetes (D_1_) and nondiabetes (N_1_) models (Table 3).

**Table 3.** Scoring systems for liver cancer prediction among patients with and those without diabetes in the absence of liver disease.

	DM^a^ (D_1_)		Non-DM (N_1_)	
Variable	Value	Point	Value	Point
Alanine aminotransferase (U/L)				
	<11.5	0	<11	0
	[11.5, 17)	2	[11, 15.5)	3
	[17, 44.4)	9	[15.5, 38)	6
	[44.4, 82.5)	24	[38, 76.3)	23
	≥82.5	29	≥76.3	29
Age (years)				
	<42	0	<39	0
	[42, 52)	12	[39, 50)	19
	[52, 72)	28	[50, 71)	29
	≥72	34	≥71	39
Sex				
	Female	0	Female	0
	Male	10	Male	6
Triglycerides (mmol/L)				
	0.735	18	<0.875	19
	[0.735, 1.03)	13	[0.875, 1.87)	13
	[1.03, 2.28)	6	[1.87, 2.83)	10
	≥2.28	0	≥2.83	0
Fasting glucose (mmol/L)				
	<6.4	0	—^b^	—
	[6.4, 9.55)	1	—	—
	[9.55, 12.8)	3	—	—
	≥12.8	8	—	—
Smoking				
	—	—	Never smoker	0
	—	—	Ever smoker	6

a DM: diabetes mellitus.

b Not applicable.

### Model Performance

The C-indexes of D_0_, N_0_, D_1_, and N_1_ on unseen test sets were 0.80 (95% CI 0.76‐0.83), 0.80 (95% CI 0.74‐0.86), 0.75 (95% CI 0.71‐0.79), and 0.82 (95% CI 0.77‐0.88), respectively. The corresponding integrated Brier scores were 0.011, 0.006, 0.011, and 0.006, respectively. 

## Discussion

### Principal Findings

This study compared risk factors, their interactions, and resulting scoring systems for liver cancer risk between patients with and those without diabetes. The resulting scoring systems took into account interaction patterns among the set of candidate predictors. Across four scoring systems stratified by diabetes and liver disease status, ALT levels, age, sex, and triglycerides were commonly chosen as predictors. Chronic viral hepatitis was uniquely selected in the overall system for diabetes. On the other hand, in the absence of liver disease, fasting glucose and smoking were uniquely chosen for diabetes and nondiabetes, respectively. Across all systems, triglycerides uniformly exhibited an inverse association with the risk of liver cancer. Among people with diabetes, chronic viral hepatitis remained the dominant risk factor for liver cancer. In the absence of chronic viral hepatitis, sex was identified as a subsequent key factor, followed by age, statin use, and ALT levels. Among people without diabetes, age became the primary factor. For older patients, statin use, LDL cholesterol, and sex became key factors when the ALT level was higher. On the other hand, smoking became a key risk factor when the ALT level was lower.

### Comparison to Previous Work

Overall scoring systems developed in this study included liver cirrhosis, ALT levels, age, and sex as common predictors across the diabetes-only and nondiabetes-only models. These variables have been commonly used as predictors of liver cancer [4,5]. Nevertheless, chronic hepatitis B or C status was uniquely chosen as a predictor for diabetes but not for nondiabetes. In the individual trees, chronic viral hepatitis only emerged as a primary factor for differentiating the risk of liver cancer in diabetes, but not in nondiabetes. Previous research suggests that hepatitis B or C infection [12,13] or hepatitis B virus (HBV)–induced cirrhosis [25] could be potentially associated with an increased risk of type 2 diabetes. Possible mechanisms include disturbances of insulin signaling pathways [12,13], alterations in glucose [12,13] and lipid metabolism [26], as well as induction of inflammation in promoting type 2 diabetes development [27]. These provide possible explanations for the more important role of chronic viral hepatitis in liver cancer development in diabetes found in this study.

In the absence of chronic viral hepatitis in diabetes, elevated ALT appeared as a key risk factor for liver cancer in females, with a higher cutoff for the younger group but a lower cutoff for the older group. While ALT is a marker for liver function [28] and a common predictor of liver cancer risk [4,5], a number of previous studies have found that ALT tends to decline with aging [14,15,29], possibly with stronger effects in males than females [16,30]. However, in this study, statin use was observed to be a more important factor in influencing the risk of liver cancer in their male counterparts. There are several possible explanations for the potential association between aging and reduced ALT thresholds for elevated liver cancer risk. In animal studies, the liver demonstrates slower and weakened regenerative ability, as well as reduced inflammatory response during aging [31]. Furthermore, in human studies, the liver appears to shrink its volume and have less blood flow during aging [32].

Moreover, in the absence of chronic viral hepatitis in diabetes, statin use emerged as a subsequent key factor in differentiating the risk of liver cancer in males, followed by age (with 51 y being symmetrically identified as optimal cutoff across statin users and non–statin users). Past observational studies have shown that statin use is potentially associated with a lower risk of liver disease [33] and liver cancer [33-35], particularly in Asian populations [34,35]. However, no effects of statin use on cancer risk have been demonstrated in randomized controlled trials [36].

Furthermore, in the presence of elevated ALT among older patients without diabetes, statin use emerged as a subsequent key factor in differentiating the risk of liver cancer, where uncontrolled LDL cholesterol and male sex were identified as dominant risk factors in statin users and nonstatin users, respectively. Previous research suggests altered lipid metabolism in carcinogenesis of the liver [26]. It is possible that under a state of potential liver dysfunction, as indicated by elevated ALT, statin use may confer protective effects against liver cancer development. Possible mechanisms of statins use against liver cancer include inhibition of tumor growth via downregulating the mevalonate pathway [37]. It has also been suggested that statins may exercise antagonistic effects against carcinogenic effects of HBV [34], potentially via suppressing cholesterol synthesis and HBV replication [38].

On the other hand, circulating triglycerides were selected as predictors and exhibited an inverse association with the risk of liver cancer across the 4 scoring systems regardless of diabetes and liver disease status. Epidemiological studies on the associations between circulating lipids and liver cancer risk remain conflicting [26,39]. Several studies found that lower levels of triglycerides [9] or total cholesterol [8,40,41] are predictive of elevated liver cancer risk in the presence [40-42] or absence [8,9] of chronic viral hepatitis. While lipids were not directly identified as a key factor in the diabetes-only individual tree, statin use emerged as a dominant factor among subgroups of patients across diabetes and nondiabetes subpopulations. Possible mechanisms linking low lipids to elevated liver cancer risk include higher lipids as indicator of preserved liver function [43], suppression of liver tumor growth via activating effector functions in natural killer cells [44], as well as uptake of circulating lipids by underlying liver tumor cells to meet their demand for lipids [39].

In addition, fasting glucose and smoking were chosen as unique predictors of liver cancer risk among patients with and those without diabetes in the absence of liver disease, respectively. The variation in baseline fasting glucose at the time of initiation of antidiabetic drugs among patients with diabetes may reflect their differential levels of diabetes severity. Previous studies have shown that fasting glucose could be individually associated with the risk of liver cancer [45]. The exact mechanism remains unknown. Possible mechanisms include upregulation of insulin-like growth factor-1 (which has a higher mitogenic and antiapoptotic potency than insulin) via hyperglycemia and hyperinsulinemia, as well as higher levels of proinflammatory factors under chronic inflammation [2,45]. On the other hand, smoking has been shown to be a carcinogenic agent to the liver in humans [46]. Several previously developed scoring systems for liver cancer risk [8,10] have also incorporated smoking as predictors. Possible mechanisms include conversion of chemicals in tobacco smoke into reactive carcinogenic metabolites in the liver, concurrent consumption of alcohol along with tobacco smoking, chronic inflammation, immunosuppression, and accelerated telomere dysfunction [47,48].

Furthermore, in the absence of diabetes, smoking emerged as a dominant risk factor for liver cancer among older patients with lower ALT. Previous research [17,18,49] suggests that smoking exposure does not directly induce liver damage, but may exacerbate liver injury caused by heavy alcohol use. Smoking was only shown to be positively associated with GGT in heavy alcohol users [17,18]. On the other hand, smoking was inversely linked to ALT and aspartate aminotransferase [17,49]. One possible explanation for elevated specific liver enzyme is that concurrent consumption of smoking and alcohol intensifies oxidative stress [50], since GGT is an oxidative stress marker [51]. In addition, previous research [52] has shown that smoking is only associated with increased ALT levels in patients who are seropositive for hepatitis C virus, but not in those who are seropositive for HBV. This may help partially explain the dominance of smoking as a risk factor among older nondiabetes patients with lower ALT from a HBV-endemic region.

### Implications

There are several public health and clinical implications of this study. First, chronic viral hepatitis appears to be a more dominant risk factor for liver cancer among patients with diabetes than those without diabetes. Second, while elevated triglycerides are one of the components of metabolic syndrome [53] and associated with adverse cardiovascular outcomes, they appear to be inversely associated with the risk of liver cancer in this study cohort. Third, in the absence of liver disease, several modifiable factors such as fasting glucose and smoking become more dominant in liver cancer risk prediction. Fourth, lipid control appears to be important to liver cancer prevention across diabetes and nondiabetes subpopulations. Fifth, the resulting linear scoring systems developed from ensemble tree-structured models reflect the interaction patterns among the set of candidate predictors in individual tree-structured models. Sixth, the risk scoring systems may help predict the risk of liver cancer in the presence or absence of liver disease across people with and those without diabetes. Further studies are needed to establish clinical thresholds to stratify patients into different levels of risk to potentially provide prioritization for public health strategies and guide clinical decision-making.

### Limitations and Future Directions

Several limitations are potentially present in this study. First, diabetes status was defined by use of antidiabetic medication. Undiagnosed diabetes or nonuse of antidiabetic medications for diagnosed diabetes is potentially present. Fasting glucose or hemoglobin A_1c_ records were not checked for potential undiagnosed diabetes cases since it was not feasible. Second, while we intend to capture an incident diabetes cohort, some patients may have received a diagnosis of diabetes before the available prescription records period. Nevertheless, delayed diagnosis and treatment initiation is likely to be partially reflected in the presence of liver disease and levels of liver enzyme and fasting glucose in the risk scoring systems. Third, the possibility of reverse causality cannot be fully eliminated. Some risk factors such as fasting glucose and triglycerides could be influenced by subclinical liver dysfunction. Altered lipid and glucose metabolism may occur under liver dysfunction [26,54]. Fourth, some potential confounders such as adiposity indicators or alcohol use were not available in this study. Fifth, current and former smoking status was not differentiated since smokers may report inconsistent status between clinical visits. Differentiating current and former smokers may further subcategorize ever smokers into 2 different risk levels in the risk scoring system for patients without diabetes in the absence of liver disease (N_1_). Sixth, information on serological tests for hepatitis virus was not available in this study. Seventh, further studies are warranted to evaluate generalizability to hepatitis C virus-endemic regions. Eighth, different populations may vary in prevalence [55], screening, diagnosis, and treatment of diabetes. Future research is warranted to examine generalizability to other populations with different diabetes management practices. Ninth, medication use and laboratory measurements were taken at baseline. However, these may change during follow-up. Tenth, the number of liver cancer cases in certain terminal nodes in the individual trees was low and may restrict generalizability of the identified optimal cutoffs beyond the study population. Eleventh, the optimal cutoffs in the scoring systems were defined by quantiles, and the values may vary across populations. Nevertheless, the optimal cutoffs could be fine-tuned [22] according to domain knowledge. Lastly, while variable selection in scoring development involves fine-tuning processes based on domain knowledge, split variable selection in separately built individual trees is automatic. Future research may consider undiagnosed diabetes, incorporate a broader list of candidate variables such as adiposity indicators and alcohol use, subcategorize current and former smoking status, evaluate serological status for hepatitis virus if available, establish clinical thresholds for risk scoring systems, and examine generalizability to other populations. 

### Conclusions

This study compared risk factors, their interaction patterns, and resulting scoring systems for liver cancer risk according to diabetes and liver disease status using tree-structured algorithms. Patients with and those without diabetes demonstrate distinctive interaction patterns among key factors on liver cancer risk. The resulting scoring systems reflect interaction patterns among the set of candidate predictors. Findings of the study may help identify targets for public health interventions and provide clinical cancer risk prediction for patients with and those without diabetes.

## Abbreviations:

ALT: alanine aminotransferase
C-index: concordance index
GGT: gamma-glutamyl transferase
HA: Hospital Authority
HBV: hepatitis B virus
HDL: high-density lipoprotein
*ICD-10*: *International Statistical Classification of Diseases, Tenth Revision*
ICPC-2: International Classification of Primary Care 2nd edition
LDL: low-density lipoprotein
